# From physical workplaces to virtual communities: extending the job embeddedness construct and predicting digital makers’ continuance participation

**DOI:** 10.3389/fpsyg.2026.1815824

**Published:** 2026-04-22

**Authors:** Zhuoying Bi, Xiao Liu, Jiaoping Yang

**Affiliations:** 1 School of Economics and Management, Qingdao University of Science and Technology, Qingdao, China; 2 School of Continuing Education, Qingdao University of Science and Technology, Qingdao, China

**Keywords:** community benefits, continuance intention to participate, emotional links, job fits, Virtual Community Job Embeddedness

## Abstract

**Introduction:**

In the digital intelligence era, makers increasingly work in virtual communities, yet it remains unclear whether traditional job embeddedness constructs apply to these new contexts. This study explores the nature of virtual community job embeddedness (VCJE) and its effect on makers’ continuance participation.

**Methods:**

Using a mixed-method design, we first applied grounded theory to interview and archival data from four leading Chinese maker communities to inductively develop the VCJE construct. We then followed a rigorous scale development procedure to produce a measurement instrument. Finally, we tested its predictive validity using survey data from 1,267 digital makers, employing hierarchical regression to examine VCJE’s impact on continuance participation intention while controlling for individual and environmental factors.

**Results:**

VCJE emerged as a three-dimensional construct comprising Community Benefits,Job Fit, and Emotional Links, each dimension differing meaningfully from traditional job embeddedness. The final 14-item scale demonstrated strong reliability and validity. Hierarchical regression revealed that VCJE significantly and positively predicts continuance participation intention, even after accounting for established predictors such as community incentives and collaboration. Among the three dimensions, Job Fit exerted the strongest influence.

**Discussion:**

This study extends job embeddedness theory into the virtual work context by redefining its core dimensions, shifting the focus from an inertial force preventing departure to a motivational force attracting continued participation. The validated VCJE scale provides a robust tool for future research on digital organizing and online labor platforms. Practically, platform operators can foster maker retention by enhancing job fit, emotional links, and community benefits. Viewed through conservation of resources and network embeddedness theories, VCJE offers a novel framework for understanding digital talent management in the Work 4.0 era.

## 1 Introduction

With the rapid development of the digital economy, the world has entered the era of digital maker economy or Work 4.0 (Skýpalová et al., 2023). This new digital work paradigm is characterized by blurred organizational boundaries, gig-based and outsourced employment models, maker-oriented work arrangements, and virtual and remote employee connections in the workplace (Chen et al., 2023). Currently, the global digital maker economy is expanding at an annual growth rate of over 25%, with its market scale exceeding US$8 trillion in 2025, emerging as a core engine driving the growth of the global digital economy. China’s digital maker sector has witnessed an even more robust development: by the end of 2024, the total number of registered digital makers on leading crowdsourcing and innovation platforms (e.g., Zhubajie and Epwk) had surpassed 120 million. This group of user include offreelancers, engineers, designers, R&D professionals, amateur innovators etc. Among them, 72% are young and middle-aged people aged 30–45, over 90% are holding college degree or above, and 68% are participating in maker activities part-time. Meanwhile, the compound annual growth rate of China’s maker communities and digital platforms has remained above 30%, with more than 5,000 new virtual maker communities established nationwide in 2024 alone. This digital maker movement has directly contributed over 1.5 trillion RMB to China’s digital economy and indirectly driven a revenue increase of more than 3 trillion RMB for upstream and downstream industries. Its value and role in technological innovation, flexible employment cultivation, and industrial structure upgrading are becoming increasingly prominent. Against this backdrop, a growing number of enterprises are shifting from traditional commodity-centric operation to community-centric management, trying to find more effective ways to build highly sticky relationships with online communities (Jones, 2023). For the digital maker communities like online crowdsourcing communities, digital innovation communities, and virtual knowledge communities, the key issue that needs to be addressed is how to improve community operation and management to attract more digital makers and gig workers and enhance their willingness to continue participating in community activities.

By definition, a digital maker community refers to the aggregate of virtual job communities and virtual organizational relationships where digital makers engage in collaborative activities. At its core lies interpersonal connections forged by shared interests and emotions, and the willingness of community members to remain long-term and maintain active participation is fundamental to the sustainable development of crowdsourcing platforms, innovation platforms, knowledge platforms, and similar digital ecosystems. In traditional work settings, Mitchell et al. (2001) found that employee turnover is influenced not only by job-specific factors but also by the social networks in which employees are embedded; they termed this intangible influence of social networks job embeddedness. Essentially, in digital work contexts such as crowdsourcing communities, a digital maker’s willingness to continuously participate in crowdsourcing projects and maintain sustained activity is shaped by both the intrinsic characteristics of the projects and the relational networks of the community. Hence, scholarly research on community structure, culture, and operation has attracted increasing attention (Jones, 2023).

In traditional work contexts, scholars have proposed constructs such as job embeddedness (Mitchell et al., 2001), career embeddedness (Ng and Feldman, 2013), family embeddedness (Purba, 2015), and team embeddedness (Chang and Cheng, 2015) to measure how employees’ organizational behaviors are shaped by their affiliated organizations, social networks, and external environments. While these constructs reflect the connection between employees and their work contexts from distinct perspectives, they all emphasize that employees and their surrounding environments form an integrated field, and many of employees’ decision-making behaviors are constrained by this field. Yang et al. (2020) argued that these different forms of embeddedness are underpinned by similar psychological mechanisms and can all be explained through three dimensions: linkages, fit, and sacrifice. This raises critical research questions: Does a similar construct exist in digital and virtual job settings, particularly in digital maker communities? Is it appropriate to directly transplant the concept of job embeddedness from traditional work settings to digital maker communities, or does the latter entail unique connotations? These questions warrant in-depth empirical and theoretical exploration.

In the digital age, the diversification, flexibility, and sharing of employment models have led to fundamental changes in both job-internal and job-external factors for many emerging occupations compared with traditional ones (Chen et al., 2023). Taking online crowdsourcing communities as an example, unlike employees in traditional workplaces, digital makers have a weak sense of hierarchical differentiation with employers, and their relationship with organizations has shifted from formal employment to contractual cooperation (Zhao et al., 2024). Many factors that constrain employee turnover in traditional settings no longer apply, meaning significant differences must exist between VCJE and the job embeddedness of traditional employees. It is therefore no longer appropriate to directly analyze digital makers’ behaviors using job embeddedness from traditional work settings and its derived constructs (e.g., organizational embeddedness, community embeddedness, career embeddedness). Meanwhile, traditional constructs such as work engagement, organizational commitment, and psychological contract primarily explain why employees “are willing to contribute” or “feel obligated to participate” from the perspective of intrinsic positive psychological states or social exchange relationships. In contrast, the unique value of job embeddedness theory lies in its emphasis on a comprehensive “binding force” or “inertial force” derived from formal and informal networks both inside and outside the organization, which explains why employees “choose to stay” or “find it difficult to leave”. In virtual job settings, the binding force of traditional bureaucratic management and physical space has declined sharply, yet informal, network-based “binding forces”—such as community relationships and emotional bonds—may instead become the key to maintaining the relationship between digital makers and platforms. This “pull mechanism” cannot be fully captured by other traditional organizational behavior constructs.

With the continuous emergence of digital and virtual scenarios, there is an urgent need to conduct in-depth analysis on how factors such as digital work contexts, community relationships, and community culture influence digital makers’ behaviors, work status, job performance, and the employer-maker relationship (Busse and Weidner, 2020; Akhavan, 2021). In particular, how to manage and maintain the relationship between enterprises and makers in digital and virtual scenarios has become an entirely new research field (González-Anta et al., 2021; Rothbard et al., 2022). To explore the impact of non-job factors in virtual spaces on digital makers’ behaviors, it is imperative to develop new variables based on job embeddedness theory. Additionally, existing constructs such as organizational embeddedness, community embeddedness, and career embeddedness fail to account for the unique forces that attract or bind individuals in virtual spaces (Jabagi et al., 2019; Wong et al., 2021). Thus, this study takes VCJE as a core construct to explore the particularities of embeddedness in digital maker communities, attempting to expand the application of job embeddedness theory in virtual job settings. Traditional job embeddedness theory is rooted in stable, face-to-face organizational environments. This study creatively introduces this theoretical framework into the context of digital and virtual maker communities to explore its applicability and evolution in the Work 4.0 era. This not only constitutes an important contextual expansion of job embeddedness theory but also a test and activation of the vitality of classic organizational behavior theories in the digital economy era.

In traditional work settings, job embeddedness and its derived constructs are often used to predict employee turnover and mobility. For comparative purposes, this study also examines the predictive effect of VCJE from the perspective of whether digital makers are willing to continue participating in community projects or exit the community. Due to the unique characteristics of digital and virtual communities (e.g., crowdsourcing communities), there is no direct equivalent to the construct of “turnover”; thus, we select its opposite—continuance participation intention—to analyze the predictive effect of VCJE on digital makers’ decision to stay or exit the community.

In summary, this study has three core research objectives: ① to develop the construct of VCJE based on grounded theory and unpack its dimensional structure; ② to compile a psychometric scale for VCJE, verify its reliability and validity through factor analysis, and provide an operable measurement tool for subsequent empirical research; ③ to validate the influence mechanism of VCJE on continuance participation intention, which also serves as a test of the construct’s predictive validity. The structure of this study draws on the general framework of similar empirical research (Hao et al., 2021; Li et al., 2021). We anticipate that the connotations of the core dimensions of job embeddedness (linkages, fit, and sacrifice) will undergo fundamental changes in virtual settings. For example, “linkages” may shift from real networks based on human, financial, and material resources to virtual networks built on psychological identification and emotional communication; “sacrifice” may transform from calculable losses of material interests to the loss of hard-to-quantify psychological benefits and a sense of community belonging. This study is the first to systematically reveal these changes and attempt to redefine the dimensions (e.g., proposing community benefits), thereby deepening our understanding of the essence of job embeddedness (the relationship between individuals and the networks in which they are embedded) and exploring its manifestations in different media environments.

## 2 Job embeddedness theory and its new expansion in virtual contexts

### 2.1 The construct of job embeddedness and its theoretical foundations

For decades, scholars have argued that employee turnover—i.e., the question of “why employees leave organizations” and “why they stay”—is primarily influenced by job attitudes, job satisfaction, organizational commitment, and job choice preferences. However, Mitchell et al. (2001) found in their empirical research that many non-job factors exhibit stronger predictive power for employee turnover. Based on this insight, they proposed the construct of job embeddedness, positing that the forces deterring employee turnover stem from two primary domains: the organization and the community in which employees live. Crucially, these forces can be distilled into three core dimensions: ① Linkages: the degree of an employee’s connectedness to other individuals and groups in the workplace and community; ② Fit: an employee’s compatibility with, and adaptability to, their work and community environments; ③ Sacrifice: the material or psychological benefits an employee would lose upon leaving their organization and community. In essence, job embeddedness is defined as a constellation of forces that prevent individuals from leaving their current jobs. Since its introduction, numerous subsequent studies have corroborated its critical predictive role in employee turnover (Purba, 2015).

Mitchell et al.’s (2001) conceptualization of job embeddedness was profoundly inspired by two foundational theories: embeddedness theory and field theory. Together, these theories frame job embeddedness as an intricate “web” that “binds” employees to their current roles and discourages turnover. The concept of embeddedness— a cornerstone of job embeddedness theory—was first introduced by the sociologist Karl Polanyi in The Great Transformation and later advanced by Mark Granovetter into embeddedness theory. This theory explains that human economic activities are not isolated but are deeply embedded in and intertwined with non-economic social networks. Field theory, also known as the “life space theory”, was proposed by the social psychologist Kurt Lewin, drawing on the physical science concept of a “field.” Lewin defined a field as a holistic configuration of the individual and their environment, encompassing both the perceptual field (physical environment) and the cognitive field (psychological environment). He argued that an individual’s motivation is jointly determined by these two interdependent fields.

When introducing the new construct of job embeddedness, Mitchell et al. (2001) also highlighted its similarities and distinctions from job attachment constructs. They posited that job embeddedness represents a novel form of organizational attachment, reflecting forces that constrain employees from leaving their current jobs. Specifically: fit resembles organizational commitment (a core component of job attachment) but excludes feelings of obligation and emotional reactivity; sacrifice aligns with continuance commitment but omits considerations of alternative options and ideological elements; linkages are analogous to supportive commitment but do not involve identification with others. Notably, job embeddedness also incorporates unique elements not captured by traditional job attachment frameworks.

Building on Mitchell et al.’s (2001) foundational work, scholars have employed diverse theoretical lenses to explain the mechanisms underlying the three dimensions of job embeddedness: ① Linkages are primarily explained by embeddedness theory. By examining social resources and individual social needs, scholars argue that the normative pressures of social networks and the constraints of social capital drive employees to remain in their current roles (Hom et al., 2012); ② Fit mechanisms draw on the Attraction-Selection-Attrition (ASA) framework and Person-Environment (P-E) fit theory (Wheeler et al., 2007; Tak, 2011). This perspective posits that compatibility between individuals and their organizations/communities fosters mutual attraction, thereby solidifying employees’ decision to stay; ③ Sacrifice is grounded in turnover theory and the investment model (Mitchell et al., 2001). It suggests that higher perceived costs of leaving (such as greater loss of resources) reduce employees’ willingness to quit. Addressing the lack of theoretical integration across these mechanisms, Kiazad et al. (2015) provided a unified explanation based on the Conservation of Resources (COR) Theory. They conceptualized linkages and fit as valuable instrumental resources for employees, while sacrifice represents valuable intrinsic resources. Leveraging COR theory, Kiazad et al. (2015) further explained the compensatory and buffering effects of job embeddedness in the workplace.

### 2.2 Expansion of the job embeddedness construct

This study argues that the classic job embeddedness construct requires targeted expansion to adapt to the characteristics of virtual job settings, with three key revisions proposed as follows:

First, the “sacrifice” dimension in classic job embeddedness can be renamed “benefit” to align its semantic connotation and emotional valence with those of “linkages” and “fit.” Since job embeddedness was initially proposed to predict turnover, it has gradually been conceptualized as a form of “inertial force” rather than a “motivational force” (Kiazad et al., 2020). Subsequent scholars have often interpreted it as the sum of forces that hinder employee turnover, rather than those that actively attract employees to stay. Although numerous studies have confirmed that job embeddedness promotes positive employee engagement and performance improvement (Liu et al., 2018), its characterization as a resistive or inertial force remains deeply entrenched. In reality, according to its theoretical foundations (including embeddedness theory, field theory, and COR theory). The forces and resources inherent in the embedded network or field are objectively described, and even framed in a positive light. Based on the COR theory interpretation of job embeddedness (Kiazad et al., 2020), we argue that the forces (or resources) depicted by job embeddedness are both inertial forces that deter leaving (resource loss) and motivational forces that attract staying (resource investment). The ultimate nature of these forces depends on the characteristics of the outcome variable being predicted, rather than the construct itself—a view supported by other scholars (Coetzer et al., 2018). Furthermore, recent research on the relationship between job embeddedness and job performance rarely invokes the “sacrifice” dimension to explain their logical connection (Chen, 2022). This is because while “sacrifice” inherently deters turnover, the absence of sacrifice does not necessarily drive work engagement. When job embeddedness is freed from its restrictive “deterrence” framework, its predictive effects on employee voice behavior, creativity, and innovation performance become more intuitive and interpretable (Ng and Feldman, 2013; Chen, 2022). Therefore, redefining the sacrifice dimension as “benefit” transcends the traditional turnover-centric, resource-loss perspective, resolving the contextual inconsistency where “linkages” and “fit” are viewed through a lens of resource conservation or investment, while “sacrifice” is viewed through a lens of resource loss.

Second, the force factors in classic job embeddedness can be appropriately expanded to include relatively enduring psychological and emotional factors. Although Mitchell et al. (2001) defined the benefits lost in “sacrifice” as encompassing both material and psychological aspects, they emphasized that the forces of job embeddedness are predominantly objective factors rather than emotional ones. In practice, however, the objective and emotional factors within an individual’s network are deeply intertwined, making it difficult for employees to disentangle them when considering turnover. Psychological and emotional resources are critical forces within work and community networks that either hinder departure or attract retention; excluding them would undermine the construct’s predictive validity. Additionally, as the theory has expanded to include constructs like family embeddedness and team embeddedness, it has become increasingly impossible to exclude emotional influences. Thus, we contend that Kiazad et al.’s (2020) reinterpretation of job embeddedness through COR theory is more robust: the work or community networks in which employees are embedded contain not only material resources but also psychological, emotional, and social resources. Yang et al. (2020) pointed out that in the Chinese context, factors such as emotional interaction and the principle of renqing (human favor) are highly significant due to the influence of guanxi (relationship) culture. In this context, it is difficult to separate the three dimensions of job embeddedness from emotions, and ignoring emotional factors would significantly reduce the construct’s predictive power. A close examination of Mitchell et al.’s (2001) foundational paper reveals that their original goal of predicting turnover led them to focus on employees’ calculations of resource gains and losses, thereby excluding temporary, impulsive, or emotional factors. However, many emotional factors are not temporary or impulsive; for instance, the need for emotional connection is a fundamental and enduring psychological need of individuals (Wang et al., 2019).

Finally, classic job embeddedness can be expanded from the absolute forces of the current job(community) to the relative forces between the current and potential new jobs(communities). While classic job embeddedness focuses on the forces of the network associated with the current job, it overlooks that employees may also be embedded in networks related to potential new jobs. In fact, research on embeddedness theory—a key theoretical source of job embeddedness—has already acknowledged the issue of dual network embeddedness. Examples include cross-regional enterprises and returnee talents who are embedded in both local and external networks (Peng et al., 2018). These studies show that dual embeddedness not only brings heterogeneous resources to focal individuals or firms but also presents challenges of choice and integration. In today’s era of high labor mobility, employees often have different places of birth, education, and employment, with family, relatives, classmates, and friends distributed across various organizations and regions. Consequently, employees may have “linkages,” “fit,” and potential “sacrifices” associated with multiple organizations and communities. An employee’s decision to leave their current organization for a new one is influenced not only by the resistive pull of the original network but also by the attractive pull of the new network. In reality, many employees leave not because the original network is weak, but because the new network exerts a stronger pull (Jia et al., 2024). From the perspective of dual embeddedness in the “turnover network” and the “recruitment network,” each network inherently contains a constellation of forces related to linkages, fit, and benefit. Whether these forces act as resistance or attraction depends entirely on the direction of the employee’s intended action.

In summary, with the expanding application of job embeddedness theory, the increasing diversification of employees’ work and lifestyles, and the construct’s inherent contextual dependence on society and culture, this paper proposes that its connotation be appropriately expanded and innovated (see Table 1).

**Table 1:** Expansion and innovation of the job embeddedness construct.

Comparison item	Classical job embeddedness	Expanded job embeddedness
Theoretical foundations	Embeddedness theory, field theory, conservation of resources theory	Embeddedness theory, field theory, conservation of resources theory
Core characteristics	Non-job forces from the individual’s network/field that influence behavior	Non-job forces from the individual’s network/field that influence behavior
Dimensional structure	Linkages, fit, sacrifice	Linkages, fit, benefit
Force factors	Objective factors	Relatively enduring factors (material, psychological, emotional, etc.)
Embedded forces	Absolute forces of a single network/field	Relative forces compared across multiple networks/fields

### 2.3 A new conceptualization of Virtual Community Job Embeddedness

A digital maker community (hereinafter referred to as the “community”) is a virtual space and a set of organizational relationships that facilitate the supply and demand of gig work in the digital economy era. Within this virtual space, traditional stable employment relationships and full-time work models no longer exist (Mulcahy, 2017), and employees are no longer required to complete work in fixed locations or at set times (Markey and McIvor, 2018). The “community” and “neighborhood” in which employees operate have broader coverage and more blurred boundaries. In this context, the formal organizations to which digital makers belong are increasingly weakened. Due to the constant change of employers, digital makers’ attachment to individual employers has shifted to attachment to digital maker platforms, and their colleagues have become all digital makers in the entire community. Consequently, the concepts of “organization” and “community” have merged into one, leading to fundamental differences in the targets and elements of “linkages,” “fit,” and “sacrifice” compared to traditional work settings.

#### 2.3.1 Linkages in Virtual Community Job Embeddedness

Digital makers’ connections with specific individuals are significantly weaker than those among members in traditional organizations or communities. First, the channels for linkages have diminished: for example, digital makers’ family members rarely interact with others in the digital maker community, and there are almost no financial connections with other individuals. Second, formal connections have been greatly reduced, with interactions primarily consisting of informal, virtual social and psychological connections, and there is almost no physical environment for in-person contact. Finally, the counterparty of the linkage has become unstable, remote, and virtual. Therefore, within the community, the strength of digital makers’ linkages with the virtual community as a space is significantly reduced. The linkage network is more of a social and psychological network, with more abstract and ambiguous targets. At this point, the force of linkage between digital makers and the community is most likely derived from social identification and psychological belonging to other community members. Relevant studies have confirmed this: community identification and belonging are important factors influencing digital makers’ continuous participation (Sun et al., 2016).

#### 2.3.2 Fit in Virtual Community Job Embeddedness

Digital makers’ adaptability and comfort within the community are not vastly different from those of traditional organizational employees in their organizations or communities. However, several critical distinctions exist: ① since digital makers’ work consists of discontinuous gig projects, they pay far less attention to long-term fit (e.g., alignment between personal values and the values of contracting enterprises); ② because crowdsourcing platforms, digital innovation platforms, and virtual knowledge platforms are virtual communities, there is no fit related to the physical environment between digital makers and the community, and psychological and emotional congruence instead becomes a core fit dimension (Xu et al., 2019); ③ due to the characteristics of the gig economy, digital makers focus more on fit in terms of working hours, personal interests, and professional abilities (Yang and Jing, 2021).

#### 2.3.3 Sacrifice in Virtual Community Job Embeddedness

The “sacrifice” dimension in traditional job embeddedness aims to measure the perceived cost of losing material and psychological benefits after turnover (Mitchell et al., 2001), such as giving up interesting projects or allowances, healthcare, pension plans, non-transferable benefits, seniority in the workplace, cultural activities provided by the community, and living welfare (Ng and Feldman, 2013; Purba, 2015). It is evident that the resources or benefits referred to in the traditional “sacrifice” dimension are relatively calculable, and the losses are substantial. In virtual job environments, compared to traditional employees, the losses digital makers incur when leaving the community are less calculable and relatively minor. First, after leaving the community, digital makers primarily lose psychological benefits, such as flow experiences, a sense of self-determination, a sense of control, accumulated fan effects, psychological capital, and data assets (González-Anta et al., 2021). Although they may also give up some material rewards, many digital makers participate in community projects in their spare time out of interest rather than as their main occupation (Jabagi et al., 2019; Acar, 2019). Even for individuals specializing in gig work, the nature of gig work results in minimal material losses (Markey and McIvor, 2018). Second, even the loss of psychological benefits is smaller than in offline environments because the switching cost to another digital maker platform is very low. The more significant losses are social-psychological and socio-emotional benefits, such as being liked or respected by community members (Han et al., 2017).

In summary, within digital maker communities, changes have occurred in the intangible networks in which digital makers’ work is embedded, the targets and elements of linkages, and the extension and categories of fit and sacrifice (see Table 2). Therefore, in digital and virtual job settings such as online crowdsourcing communities, digital innovation communities, and virtual knowledge communities, it is necessary to innovate and develop the concept of job embeddedness from traditional work settings.

**Table 2:** Comparison between traditional job embeddedness and VCJE.

Comparison item	Traditional job embeddedness	Virtual community Job embeddedness
Source of force	Relatively stable work relationships, organizational environment, or living community.	Virtual job environment, contractual work relationships, and boundaryless network communities.
Linkage dimension	Connection channels such as human, financial, and material resources; strong ties; real linkage targets.	Connection channels such as psychological and emotional bonds; weak ties; abstract linkage targets.
Fit dimension	Physical fit, value fit, interest-ability fit, etc.	Psychological fit, emotional congruence, interest-ability fit, etc.
Sacrifice dimension	Calculable material rewards, objective benefits, and tangible losses.	Psychological and emotional gains, virtual benefits, and psychological losses.

## 3 Construction of Virtual Community Job Embeddedness dimensions based on grounded theory

### 3.1 Theoretical sampling

This study selected digital makers from four platforms—Zhubajie.com, Epwk.com, Xiaomi Community, and Haier HOPE Community—as interview subjects. Among them, Zhubajie.com and Epwk.com are professional third-party crowdsourcing service platforms, ranking second and third among China’s professional crowdsourcing platforms, respectively. Xiaomi Community and Haier HOPE Community are virtual innovation communities led by manufacturing enterprises, which are highly representative in their category. Overall, these four digital maker platforms cover most types of major digital maker platforms in China. Meanwhile, these digital maker communities are mature and well-operated, meeting the theoretical sampling requirements of grounded theory.

To obtain richer interview data and considering information volume and variability, we referenced the demographic distribution of Chinese netizens from the 55th CNNIC (China Internet Network Information Center) Statistical Report and the research on digital makers by other scholars (Ma et al., 2018) to set the following criteria for interview subjects: ① holding a college degree or above;② having undertaken part-time work, gig work, or innovation projects on relevant platforms for more than one year; ③ having participated in the current digital maker community for more than six months;④ no more than 20% of subjects being from the same province; ⑤ the proportion of female subjects not less than 40%. Based on these criteria, 58 digital makers were ultimately selected.

### 3.2 Data collection

Grounded theory allows for multiple sources of analytical data. This study integrated in-depth interviews, online materials, and literature to collect required data, with the data collection period spanning from August 2020 to August 2023. First, 28 digital makers were randomly selected from the 58 subjects for one-on-one interviews (either online or face-to-face). Professors and practitioners in the fields of innovation management and human resource management were invited to join the interview team. Before each interview, subjects were briefed on the research purpose and related content. For the 20 digital makers interviewed online, each session lasted no less than 30 min; for the 8 face-to-face interviews, each session lasted no less than 50 min. The interviews focused on questions such as: “Please introduce the digital maker community you frequently participate in; why are you willing to continuously participate in crowdsourcing (part-time/gig) projects on this community; what do you think are the biggest differences between working in a digital maker community and an offline work environment; if you had to leave this platform or community, what would you be reluctant to give up?”

Second, to ensure the validity and comprehensiveness of the research, the remaining 30 digital makers were randomly divided into 3 groups, and 3 rounds of Focus Group Discussion (FGD) were conducted around the above outline. Finally, referring to the research method of Li et al. (2021), literature on crowdsourcing published on CNKI (China National Knowledge Infrastructure) and online reports on crowdsourcing and gig platforms were used as supplementary qualitative data. The first economics and management paper on crowdsourcing published in core journals and CSSCI journals on CNKI dates back to 2017; thus, the literature search spanned from 2017 to 2025, with retrieval keywords including “crowdsourcing, innovation community, and virtual community.” A total of 134 articles and reports were selected after screening.

### 3.3 Coding process

#### 3.3.1 Open coding

In the open coding stage, first, the interview data were independently coded by two groups. During the initial coding, we prioritized using the interviewees’ own words or phrases to ensure an authentic reflection of the characteristics of VCJE. Next, similar codes were merged, and conflicting codes were eliminated to obtain the final coding results for each group. A comparison of the two groups’ results showed a coding consistency of 86.36%. Finally, the two groups cross-checked the coding process and conducted collective discussions, ultimately identifying 63 initial concepts (see Table 3).

**Table 3:** Examples of open interview concept coding.

Original interview excerpts	Conceptual coding	Frequency
“I am an engineering major and enjoy hands-on operations (a11). I usually like to disassemble, assemble, and even design small things myself. The community happens to provide a platform (a12). Moreover, chatting with other community members about interesting topics (a14) makes me feel relaxed. I like to join in the fun (a15); especially when I’m bored, I often visit the community to see if there’s anything interesting (a16).”	a11 Vocational interest	15
	a12 Provision of platform	6
	a13 Common topics	13
	a14 Mood relaxation	9
	a15 Entertainment orientation	8
“I really like Xiaomi’s products (a21); they are fashionable and easy to use, matching my aesthetic (a22). Especially since many community members also like Xiaomi’s products—we share similar values and get along well (a23). I am willing to post in the community (a24), and many of my high-quality posts have received attention (a25). I think my views are somewhat insightful (a26).”	a21 Demand preference	13
	a22 Alignment with hobbies	12
	a23 Congruent values	8
	a24 Expression of opinions	14
	a25 Receiving attention	13
	a26 Gaining recognition	10
“In my spare time, I can use my professional expertise (a31) to provide technical solutions for enterprises or projects, earning extra income (a32) and realizing the value of my professional knowledge (a33). Through communication and interaction with other community members (a34), I can learn a lot of information and skills that are helpful for my work and life (a35). Most of the time, I chat happily with other members in the community (a36), sharing joys and sorrows (a37), which makes me feel the warmth of home (a38).”	a31 Utilization of expertise	12
	a32 Earning income	16
	a33 Realization of value	12
	a34 Social exchange	10
	a35 Obtaining assistance	7
	a36 Feeling happy	6
	a37 Mutual sharing	12
	a38 Warm atmosphere	9

#### 3.3.2 Axial coding

Axial coding involved screening, merging, and categorizing the 63 initial concepts to form the subcategories and main categories of grounded theory, based on the logical relationships between concepts (e.g., causal, contextual, and hierarchical relationships). After strict consistency checks, 13 subcategories and three main categories were finalized (see Table 4).

**Table 4:** Main categories, sub-categories, and conceptual coding.

Core category	Main category	Sub-category	Conceptual coding
Virtual Community Job Embeddedness	Community benefits	Value realization	Provision of platform; expression of opinions; receiving attention; demonstration of talents
		Self-development	Obtaining assistance; acquiring knowledge; gaining information; receiving training; self-improvemen
		Material gains	Earning income; winning rewards; supplementing household expenses; obtaining discounts
		Spiritual satisfaction	Mood relaxation; gaining recognition; feeling happy; enhancing prestige; acquiring friendship; winning respect
	Job fit	Interest fit	Vocational interest; alignment with hobbies; leisure hobbies; work enjoyment; work excitement
		Supply–demand fit	Demand preference; reasonable reward for effort; abundant projects; effective incentives; extensive participation
		Value fit	Entertainment orientation; shared pursuit; emphasis on ability; realization of value; fulfillment of ideals; contributing to society
		Ability fit	Utilization of expertise; ease in performing tasks; professional proficiency; leveraging strengths; stimulating potential
		Method fit	Time flexibility; spatial flexibility; flexible working methods; utilization of leisure time
	Emotional links	Social connections	Social exchange; community circles; organizing activities; helping others
		Positive atmosphere	Mutual sharing; warm atmosphere; harmonious relationships; equality; mutual tolerance; openness and transparency
		Community belonging	Common topics; congruent values; sense of security; active participation; virtual friendship
		Community communication	Shared interests; open expression; willingness to listen; emotional encouragement

#### 3.3.3 Selective coding

Selective coding focused on analyzing the internal logical relationships between main categories and subcategories to identify and confirm the core category from existing categories. On the one hand, we analyzed the connotation and conceptual boundary of each category; on the other hand, we compared each category with the classic dimensions of job embeddedness (Mitchell et al., 2001) to clarify their differences and connections. The three main categories were interpreted as follows:

Community benefits: As shown in Table 2, this category primarily reflects the material and psychological gains digital makers obtain from the community, which is most aligned with the “sacrifice” dimension in traditional job embeddedness. However, since participation in crowdsourcing projects is mostly part-time or leisure-based, the loss incurred by leaving the community is significantly smaller compared to traditional work settings. Thus, from the perspective of “community benefits,” the force of VCJE that “retains” digital makers and prevents them from leaving is relatively weakened (Yang and Jing, 2021).

Emotional links: This category expresses the closeness of digital makers’ connections to the community and their sense of belonging. It is most analogous to the “linkages” dimension in traditional job embeddedness. Nevertheless, unlike traditional work settings, digital maker communities emphasize digital makers’ connections to the broader virtual field rather than individual-specific ties, which are greatly reduced. Additionally, in virtual environments, digital makers are more likely to find like-minded groups, leading to deeper emotional exchanges, even immersion and attachment (Jones, 2023). Therefore, from the perspective of “emotional links,” the strength and focus of the retaining force of VCJE have increased and shifted compared to traditional contexts.

Job fit: This category reflects the degree of alignment between community-provided tasks and digital makers’ interests/abilities, as well as the congruence of values among members. It is essentially consistent with the “fit” dimension in traditional job embeddedness. However, interviews clearly revealed that digital makers perceive easier achievement of supply–demand fit, value fit, method fit, and ability fit in virtual environments—explaining why many digital makers participate in crowdsourcing projects not solely for material benefits (Acar, 2019).

Given the significant differences between “community benefits, job fit, and emotional links” and the traditional job embeddedness dimensions of “sacrifice, fit, and linkages,” the core category was identified as VCJE.

### 3.4 Results validation

To ensure the research reliability, the coding process was independently conducted by 6 trained master’s and doctoral students, with 2 professors specializing in human resource management invited to review the coding procedure. The consistency of the coding results between the two groups exceeded the 80% threshold (Li et al., 2021), indicating satisfactory reliability.

To guarantee the research validity, two measures were implemented: first, interview subjects were strictly screened based on criteria including: ① participation in the community for more than 1 year; ② engagement in the current digital maker community for over 6 months; ③ holding a college degree or above; ④ no more than 20% of subjects from the same province; and ⑤ a female proportion of no less than 40%. Second, 134 journal articles and online materials were incorporated as supplementary qualitative data for coding analysis, maximizing the validity of the research.

To test theoretical saturation, a comparison was conducted between the interview data of the first 28 digital makers and the latter 30 digital makers. No new important categories emerged from the subsequent interviews, demonstrating that the research results have reached theoretical saturation.

### 3.5 Results analysis

Based on the Conservation of Resources (COR) Theory, the three main categories of VCJE extracted in this study can be analyzed as follows: emotional Links: Similar to the “linkages” dimension in traditional job embeddedness, this category explores individuals’ social needs and motivations for acquiring social resources, and can also be interpreted using embeddedness theory and COR theory. The key difference lies in that the objects of social connection in virtual contexts are relatively broader and more abstract. Job Fit: Essentially consistent with the traditional “fit” dimension, this category examines the compatibility between individuals and their environment, ensuring that their resources are effectively utilized without loss. Community Benefits: Discussed from the opposite perspective of resource loss, this category represents valuable resources cherished by individuals and can be explained using the investment model. While it shares an inherent connection with the traditional “sacrifice” dimension, it differs in perspective and intensity. Additionally, the context of VCJE has shifted from the dual contexts of “organization and community” in traditional job embeddedness to a single “community” context. Specifically, the boundarylessness of virtual environments has weakened organizational characteristics.

Compared with traditional job embeddedness, VCJE places significantly greater emphasis on psychological and emotional forces. Traditional job embeddedness was initially proposed to predict turnover, thus emphasizing its function of deterring turnover. Although it acknowledges the existence of subjective psychological and emotional forces in deterring turnover, it assumes that employees primarily consider objective forces (Mitchell et al., 2001). In virtual environments, however, the concept of “turnover” has become blurred, leading to a decline in the influence of objective forces and a rise in subjective ones. In fact, research based on COR theory argues that job embeddedness reflects a state where employees possess abundant emotional attachment resources and a strong sense of organizational identification (Kiazad et al., 2020)—a view that explicitly affirms the psychological and emotional components. Thus, VCJE does not deviate from the overall framework of job embeddedness but rather represents a context-specific form of it.

By renaming the traditional “sacrifice” dimension as “community benefits,” VCJE ensures that all three dimensions interpret the resources empowered by the embedded work or community network from a positive perspective for digital makers (or employees). When digital makers choose to stay in the community, these resources facilitate their future resource investment and motivate proactive behaviors; when they consider leaving, the potential loss of these resources increases their opportunity cost of departure.

As an important theoretical foundation of job embeddedness, classical embeddedness theory classifies “embeddedness” into two forms: relational embeddedness and structural embeddedness. Focal individuals or organizations in embedded networks can acquire social capital through relational or structural embeddedness (Yuksel et al., 2019). Thus, “relational embeddedness” and “structural embeddedness” can be regarded as the state dimensions of network embeddedness, while “social capital” is its outcome dimension. Within the framework of classical embeddedness theory, the “linkages” and “fit” dimensions in traditional job embeddedness serve as state dimensions (analogous to relational and structural embeddedness), and “sacrifice” acts as the outcome dimension (with its opposite perspective analogous to social capital). Similarly, in VCJE, “emotional links” and “job fit” are state dimensions, and “community benefits” is the outcome dimension.

In summary, VCJE is a unique structure of job embeddedness. Drawing on the definition proposed by Halbesleben and Wheeler (2008), it can be defined as the force that drives digital makers’ willingness to continuously participate in digital maker communities, rooted in their abundant emotional attachment resources and strong sense of organizational identification with the community.

## 4 Development of the Virtual Community Job Embeddedness scale

### 4.1 Item collection for Virtual Community Job Embeddedness

The overall scale development process is as follows: first, the definition of the construct was presented to the participants; second, open-ended questionnaires were used to collect participants’ behavioral descriptions, which were then categorized and statistically analyzed as item materials; finally, important items were selected based on frequency and representativeness to form a test item pool for each dimension.

The participants of the open-ended questionnaire survey were digital makers from Zhubajie.com and Haier Hope Platform. After communication, 66 digital makers agreed to participate in the study. Among them, 39 were male and 27 were female, with an age range of 24 to 45 years old. Their experience in participating in platform-commissioned projects ranged from 1 to 5 years. Except for 7 participants with a high school education, all others held a college degree or above. Among the participants, 19 were freelancers, and the remaining digital makers worked in various industries including education, finance, information technology, manufacturing, and healthcare.

The collected questionnaires were processed as follows: first, each participant’s statements were semantically segmented (similar to the labeling process in grounded theory) to ensure that each segmented label represented an independent meaning; second, all segmented labels were classified into three dimensions according to the three types of attachment relationships: community benefits, job fit, and emotional links; third, labels with similar content were summarized into multiple subcategories; finally, similar labels and subcategories were merged and deleted to obtain the materials for the VCJE scale.

A total of 331 label contents were segmented from the statements of 66 digital makers. Two researchers independently classified these labels, and 35 labels with inconsistent classifications were deleted. The label contents of each subcategory were summarized (similar to initial coding in grounded theory), resulting in the results shown in Table 5.

**Table 5:** Main categories, subcategories, and corresponding items.

Main category (dimension)	Subcategory (item)	Representative statement	Number of labels
Community benefits	VB1 Obtaining material or financial rewards	“I can get price discounts when purchasing products of relevant brands”	23
	VB2 Gaining status or prestige	“I am quite popular in the community and have many followers”	7
	VB3 Obtaining training or ability improvement	“I have acquired a lot of new knowledge”	19
	VB4 Gaining the pleasure of free expression	“I can independently express my views and opinions”	6
	VB5 Gaining recognition and affirmation	“This makes me feel that my ideas are valuable”	11
	VB6 Achieving joy or positive experiences through participation	“I can gain a lot of joy in this community”	18
	VB7 Gaining friendship through community communication	“I met new friends by participating in this project”	17
Job fit	VF1 Matching between projects and skills/talents	“I can display my skills and talents in this community”	19
	VF2 Contributing to the realization of ideals	“It fulfilled my childhood dreams”	13
	VF3 Matching between rewards and efforts	“The hard work I put in can get reasonable rewards”	21
	VF4 Temporal and spatial matching of work methods	“It fits my schedule and work style”	27
	VF5 Suitability of the community’s cultural atmosphere	“Communication in the community is very relaxed and pleasant”	8
	VF6 Matching between work and interests/hobbies	“I like this type of work quite a lot”	13
Emotional links	VL1 digital makers with similar interests in the community	“There are many people in the community who share similar interests with me”	7
	VL2 Netizens with consistent values in the community	“We can share joys and sorrows with each other”	13
	VL3 Close friends with frequent interactions in the community	“I often keep in touch with several good friends”	5
	VL4 Harmonious relationships with many community members	“I have harmonious relationships with many members in the community”	18
	VL5 Open and sincere communication among community members	“Everyone’s communication is based on equality”	5
	VL6 Friends with common topics in the community	“I get along very well with several friends and have a lot to talk about”	12
	VL7 The community feels like a big family	“It makes me feel the warmth of a family”	34

### 4.2 Compilation of the Virtual Community Job Embeddedness scale

First, a literature search was conducted using Chinese and foreign databases, including CNKI (China National Knowledge Infrastructure), Web of Science Core Collection, and EBSCO. By reviewing existing literature, constructs similar to community benefits, job fit, and emotional links (such as virtual community belonging, emotional attachment, and community involvement) were extracted, and relevant scale items that could be integrated into the three dimensions of VCJE were selected.

Second, drawing on traditional job embeddedness scales, relevant items were contextually adapted to serve as one of the sources of initial items for the scale developed in this study. This is because, according to the conceptualization of VCJE developed through grounded theory analysis, its three dimensions—emotional links, job fit, and community benefits—are a context-specific structure of the three dimensions of job embeddedness (linkages, fit, and sacrifice), and the two constructs share similarities and connections in many aspects.

Finally, based on Table 4, the outlines of each item (subcategory codes) were deduced in accordance with the expression style of scales to form test items. The items obtained through the above three steps were further compared and analyzed, and items with essentially duplicate or similar meanings were eliminated, resulting in a total of 37 items. Among them: Community Benefits (denoted as VEB) includes 14 items (VB1 ~ VB14); Job Fit (denoted as VEF) includes 10 items (VF1 ~ VF10); Emotional Links (denoted as VEL) includes 13 items (VL1 ~ VL13).

### 4.3 Pre-test and item purification

First, a pre-survey was conducted on Zhubajie.com and Haier HOPE Innovation Platform via Wenjuanxing (a professional online survey platform). A total of 363 questionnaires were collected, and after excluding those with obvious response patterns and missing data, 258 valid questionnaires were obtained, with an effective response rate of 71.1%. A mean comparison test showed no significant demographic differences between invalid and valid questionnaires, indicating no obvious non-response bias.

The demographic characteristics of the pre-survey sample were as follows: 74.2% were male; 65.7% were aged 21–30 years, and 22.7% were aged 31–40 years; 171 respondents (66.3%) were unmarried; 22.1% had an education level of college or below, 50.8% had a bachelor’s or master’s degree, and 27.1% had a doctoral degree; 60.9% had participated in community-related activities for less than 1 year, 22.5% for 1–3 years, and 16.6% for more than 3 years.

The item purification process proceeded in five steps: ① Critical Ratio (CR) Analysis: Items with non-significant critical ratios were eliminated, including 12 items: VB4, VB9, VB12, VB14, VF2, VF7, VF10, VL5, VL8, VL9, VL11, and VL13. ② Inter-item Correlation Check: Items with absolute correlation coefficients less than 0.5 with all other items were removed, resulting in the deletion of 5 items: VB10, VB13, VF5, VL1, and VL12. ③ Reliability Test: The consistency of the remaining 20 items was assessed. The Cronbach’s *α* coefficients for the three dimensions of VCJE were 0.836, 0.876, and 0.845, with an overall scale α coefficient of 0.817—all exceeding the 0.7 threshold, indicating satisfactory reliability. ④ Factor Analysis with Orthogonal Rotation: Principal component analysis (PCA) with orthogonal rotation was conducted. Items with communalities below 0.4 (VB2, VB11, VF8, VF9) and items with cross-loadings (VB5, VL3) were eliminated. ⑤ Exploratory Factor Analysis (EFA): EFA was performed on the final 14 items (see Table 6). The KMO value was 0.843, and Bartlett’s Test of Sphericity was significant (*p* < 0.001), confirming that the data were suitable for factor analysis.

**Table 6:** Virtual Community Job Embeddedness scale and exploratory factor analysis results.

Dimension	Item	Component		
		Component 1	Component 2	Component 3
Community benefits (VEB)	VE3: I can gain a lot of joy in this community	**0.805**	0.276	0.215
	VE1: I can receive remuneration commensurate with my work in this community	**0.804**	0.253	0.307
	VE5: I have gained friendship in this community	**0.801**	0.313	0.230
	VE4: I have acquired a great deal of knowledge since joining this community	**0.782**	0.294	0.260
	VE2: Participating in gig projects in this community will be helpful for my future	**0.776**	0.221	0.226
Emotional links (VEL)	VE12: There are community members with whom I share common topics	0.234	**0.810**	0.288
	VE10: There are many friends in this community with consistent values	0.283	**0.802**	0.244
	VE11: I have harmonious relationships with many community members	0.262	**0.785**	0.225
	VE14: There are many community members willing to share joys and sorrows with me	0.260	**0.779**	0.226
	VE13: I sometimes feel that this community is like a family	0.295	**0.771**	0.223
Job fit (VEF)	VE8: The projects in this community are highly compatible with my interests	0.256	0.174	**0.815**
	VE6: I can display my skills and talents in this community	0.312	0.261	**0.780**
	VE7: The projects in this community fit my schedule and work style	0.192	0.321	**0.776**
	VE9: I feel more able to realize my value in this community	0.277	0.283	**0.760**

Bold values indicate the factor loadings of each item on its primary factor (i.e., the factor to which the item is assigned). Non-bold values represent cross-loadings on other factors.

Using SPSS 22.0, PCA with orthogonal rotation extracted 3 component factors, with a cumulative variance explained of 75.810%. All factor loadings exceeded 0.760 (see the rightmost columns of Table 6).

Component 1: Corresponding items primarily reflect digital makers’ material and psychological gains in the community, aligning with the “community benefits” category derived from grounded theory. This dimension is most analogous to the “sacrifice” dimension in traditional job embeddedness. However, since participation in community projects is mostly part-time or leisure-based, the potential loss of benefits upon leaving the community is significantly smaller compared to traditional work settings.

Component 2: Items in this factor reflect the closeness of digital makers’ connections to the community and their socio-psychological and emotional sense of belonging. Corresponding to the “emotional links” category, it is analogous to the “linkages” dimension in traditional job embeddedness. Unlike traditional work settings, however, digital maker communities emphasize digital makers’ connections to the broader virtual field rather than individual-specific ties (which are greatly reduced), and these connections are primarily socio-emotional.

Component 3: Items in this factor reflect the alignment between community-provided tasks and digital makers’ interests/abilities, as well as value congruence among members. Corresponding to the “job fit” category, it is essentially consistent with the “fit” dimension in traditional job embeddedness.

## 5 Validation of the Virtual Community Job Embeddedness scale

### 5.1 Reliability and validity tests

Questionnaires were distributed to relevant digital makers on two crowdsourcing platforms, Zhubajie.com and Epwk.com, with the assistance of a research company. A total of 1,493 questionnaires were collected, of which 1,267 were valid, yielding an effective response rate of 84.86%. To verify the factor structure of VCJE, Table 7 compares the goodness of fit of the three-factor model with that of other alternative models. As shown in Table 7, the three-factor model exhibits the best goodness of fit among all models, which further demonstrates good discriminant validity among the variables. Thus, the three-dimensional structure of VCJE is accepted.

**Table 7:** Confirmatory factor analysis of Virtual Community Job Embeddedness.

Model	χ^2^/Df	RMSEA	SRMR	TLI	CFI	NFI
Single-factor	25.831	0.140	0.077	0.763	0.799	0.764
Two-factor a	13.226	0.098	0.055	0.883	0.902	0.867
Two-factor b	13.525	0.099	0.056	0.880	0.900	0.862
Two-factor c	16.512	0.111	0.061	0.852	0.876	0.837
Three-factor	1.219	0.013	0.014	0.998	0.998	0.963
Reference criteria	<3.0	<0.1	<0.1	>0.90	>0.90	>0.90

Two-factor a: VEL + VEF, VEB; Two-factor b: VEL + VEB, EBF; Two-factor c: VEL, VEF + VEB.

The composite reliability, correlation coefficients, and average variance extracted (AVE) values of each latent variable are presented in Table 8. It can be seen that the correlation coefficients among the three internal dimensions of VCJE are all moderate to high; the correlation coefficient between each pair of dimensions is smaller than the square root of the AVE of the corresponding dimensions, indicating good discriminant validity among the dimensions. In addition, the results of the confirmatory factor analysis (CFA) show that all dimensions exist independently and do not need to be merged despite the aforementioned correlations. Both the Cronbach’s *α* coefficients and composite reliability (CR) values are above 0.70, which indicates that the questionnaire has good predictive stability and structural reliability.

**Table 8:** Correlation, reliability and validity analysis of Virtual Community Job Embeddedness.

Variables and Indicators	Job fit	Emotional links	Community benefits
Job fit	(0.770)		
Emotional links	0.605^***^	(0.768)	
Community benefits	0.617^***^	0.623^***^	(0.784)
Mean	3.975	3.948	3.979
Standard deviation	1.156	1.182	1.178
Composite reliability (CR)	0.853	0.878	0.888
Cronbach’s α	0.853	0.878	0.888

***p < 0.01; the numbers in parentheses are the square roots of AVE. The same applies to the subsequent tables.

### 5.2 Test of predictive effects

Based on the analysis above, job embeddedness, especially VCJE, can be regarded as both a resistance to turnover and an attraction to retention. However, “digital maker turnover” in virtual environments is inherently different from “employee turnover” in traditional settings. Furthermore, “digital maker turnover” is not a primary concern in open environments such as crowdsourcing platforms and virtual communities. Therefore, taking crowdsourcing communities as an example, this section uses the commonly adopted concept of “continuance intention to participate” instead of “digital maker turnover” to test the predictive validity of VCJE.

According to existing research (Wang et al., 2020), digital makers’ individual characteristics influence their continuous participation intention. Thus, gender, age, and education level were selected as individual-level control variables. Additionally, the supportive and controllable environment of crowdsourcing communities is a crucial factor affecting participation intention. Relevant studies have also indicated that the incentive mechanisms and collaboration mechanisms of crowdsourcing communities help enhance digital makers’ willingness to participate (Sun et al., 2016). Therefore, this study adopts two important dimensions of community governance—“community incentives” and “community collaboration”—as environmental-level control variables.

Conservation of Resources (COR) Theory and Network Embeddedness Theory are the two key theoretical foundations of VCJE. Network Embeddedness Theory describes the state of embeddedness and the outcomes obtained after embedding (Yang et al., 2020), while COR Theory explains the intentions, motivations, and behaviors of digital makers or employees post-embedding (Kiazad et al., 2015). Specifically, Network Embeddedness Theory addresses the source of resources, and COR Theory addresses the management of resources. The following analysis examines the impact of VCJE on digital makers’ continuous participation based on COR Theory.

#### 5.2.1 Research hypotheses

From the perspective of Community Benefits, when digital makers continuously participate in crowdsourcing communities and undertake projects, they can obtain material rewards (Han et al., 2017). Completing projects independently or collaboratively, as well as interacting with community members, allows them to acquire knowledge, accumulating capital for future competence enhancement and career development (Acar, 2019). Simultaneously, participating in community interactions and undertaking crowdsourcing projects helps them gain friendship and satisfy personal interests (Amor et al., 2021). Once they leave the community, however, some material or non-material resources obtained therein will either cease to appreciate or be lost entirely. Therefore, unless leaving the community yields more resources, digital makers will be willing to continue their participation. Accordingly, the following hypothesis is proposed:

*H1*: digital makers’ perceived community benefits in a crowdsourcing community have a positive impact on their continuous participation intention.

From the perspective of Job Fit, first, when digital makers participate in crowdsourcing communities and secure projects they enjoy or excel at, it helps stimulate their interest, leverage their skills and talents, and enhance their sense of satisfaction and competence (Yang and Jing, 2021). Once digital makers gain these valuable psychological resources (satisfaction and competence), they will feel energized, willing to continue completing projects, and motivated to preserve these resources through sustained participation. Second, when digital makers perceive that they can realize their self-worth (Yang and Jing, 2021) and gain important psychological resources such as social presence and self-affirmation (Acar, 2019) within the community, they will also be willing to continuously participate to protect and even augment these resources. Accordingly, the following hypothesis is proposed:

*H2*: digital makers’ perceived job fit in a crowdsourcing community has a positive impact on their continuous participation intention.

From the perspective of Emotional Links, first, frequent and harmonious interactions, as well as mutual trust and recognition among digital makers, foster emotional attachment and a sense of belonging (Wang et al., 2020). On the one hand, this emotional attachment and belongingness are important psychological resources for digital makers. On the other hand, a sense of belonging to the online community helps form a more harmonious atmosphere, enabling access to more resources such as information and experience (Vohra and Bhardwaj, 2019). To preserve these resources, digital makers will be willing to continue participating in the crowdsourcing community. Second, when digital makers have numerous online friends in the community who share common topics and similar values, their identification with other community members increases (Han et al., 2017), thereby enhancing their psychological resources. This identification facilitates the formation of reciprocal relationships (Ma et al., 2018), which in turn help obtain more material and non-material resources, motivating digital makers to sustain their participation. Accordingly, the following hypothesis is proposed:

*H3*: digital makers’ perceived emotional links in a crowdsourcing community have a positive impact on their continuous participation intention.

From the perspective of the overall construct of VCJE: first, the material and non-material resources gained through embeddedness enhance digital makers’ self-efficacy. Mutual trust and reciprocal norms formed during the embedding process continuously strengthen digital makers’ collective consciousness and psychological ownership of the crowdsourcing community (Ma et al., 2018), thereby increasing their willingness to contribute continuously. Second, according to COR Theory, when VCJE brings resources such as information, knowledge, material benefits, emotional support, and even psychological recognition, digital makers will view these as valuable resources to be preserved, striving to avoid their loss. Consequently, they will not easily leave the community and will instead continue participating to initiate a resource gain spiral, thereby acquiring more resources. Finally, as digital makers become tightly embedded in the virtual network of the crowdsourcing community, increased communication and interaction with others will gradually foster emotional attachment (Wang et al., 2020), a sense of belonging to the community as a whole, and over time, emotional involvement (Zhou and Wu, 2025). They will perceive themselves as insiders and owners of the community, thus willing to participate continuously. Based on the above analysis, the following hypothesis is proposed:

*H4*: VCJE has a positive impact on digital makers’ continuous participation intention.

#### 5.2.2 Questionnaires and data

With the exception of Virtual Community Job Embeddedness (denoted as VCJE), all other scales adopted mature measurement tools from domestic and international studies, with appropriate revisions tailored to the crowdsourcing context. The details are as follows: ① Community motivation (VMO) and community collaboration (VSY) were revised based on the scale developed by Gu et al. (2019), consisting of 5 and 4 items respectively; ② Digital makers’ continuance intention to participate (CPI) was measured using a 3-item scale from the study of Wang et al. (2020). Among the 1,267 valid questionnaires, 76.7% were completed by male digital makers; 90.4% of the respondents were aged 21–35 years; 59.9% were unmarried; 93.4% held a college degree or above; 44.6% had less than 1 year of community participation experience, and 15.9% had more than 3 years of experience.

Common method bias was tested using Harman’s single-factor test and the unmeasured latent method factor test. The Harman’s single-factor test showed that the first unrotated factor extracted from exploratory factor analysis explained 35.86% of the total variance, accounting for less than half of the total explained variance. For the unmeasured latent method factor test, the average variance extracted (AVE) of the common method variance latent factor was 0.31 after its inclusion in the model, which is below the critical threshold for identifying significant common method bias. As shown in Table 9, the Cronbach’s *α* coefficients and composite reliability (CR) values of all 6 construct dimensions exceeded 0.8, and their AVE values were all above 0.5.

**Table 9:** Results of factor analysis.

Variable	Construct dimension	Factor loading	Cronbach’s α	CR	AVE
Virtual Community Job Embeddedness	Emotional links	0.735–0.815	0.853	0.853	0.593
	Job fit	0.733–0.794	0.878	0.878	0.590
	Community benefits	0.762–0.801	0.888	0.888	0.614
Community motivation		0.735–0.800	0.880	0.880	0.595
Community collaboration		0.760–0.814	0.889	0.890	0.619
Continuance intention to participate		0.748–0.828	0.841	0.841	0.621

Table 10 reports the correlation coefficients among the latent variables. By comparing the square root of the AVE of each variable with its correlation coefficients with other variables, it was confirmed that all variables had good discriminant validity. These results also indicated that VCJE is a distinct construct from community motivation and community collaboration. In summary, the scales demonstrated good reliability and validity.

**Table 10:** Correlation matrix of latent variables.

Variable	VCJE	MO	SY	CPI
Virtual Community Job Embeddedness	(0.839)			
Community motivation	0.301^**^	(0.771)		
Community collaboration	0.227^**^	0.613^**^	(0.787)	
Continuance intention to participate	0.592^**^	0.585^**^	0.597^**^	(0.788)
Mean	3.967	3.989	3.997	4.696
Standard deviation	1.051	1.173	1.190	0.999

**p < 0.05.

VCJE and continuance intention to participate were individual-level variables (at the digital maker level), while community motivation and community collaboration were contextual variables (at the community level). All data were collected from individual digital makers. The r_wg values of community motivation and community collaboration were 0.681 and 0.634, respectively, both below the 0.7 threshold. Their ICC (Acar, 2019) values were 0.071 and 0.073, indicating a moderate within-group correlation, and thus multilevel analysis was deemed unnecessary. The lack of support for aggregating the two community-level variables to the individual level may be attributed to two reasons: first, although the survey was conducted across four communities, each community contained multiple sub-modules with significant internal heterogeneity; second, digital makers exhibited substantial differences in their demands and perceptions, leading to divergent evaluations of the same community mechanisms.

#### 5.2.3 Hypothesis testing

As shown in Table 11: After adding the explanatory variable VCJE to the control variables in Model 2, the model shows that the coefficient for VCJE is significantly positive (*β* = 0.304, *p* < 0.01). This indicates that VCJE has a significant impact on digital makers’ continuous participation intention. In particular, as shown in Model 3, when the effects of community incentives and community collaboration are not considered, the impact of VCJE on continuous participation intention is even stronger (*β* = 0.593, *p* < 0.01), thus verifying Hypothesis H4.

**Table 11:** Hierarchical regression analysis.

Variables	Digital makers’ continuous participation intention			
	Model 1	Model 2	Model 3	Model 4
Control variables				
Digital maker gender	−0.039	−0.035	−0.051	−0.030
Digital maker age	−0.004	−0.032^**^	−0.034^**^	−0.012
Participation tenure	0.019	0.012	0.020^*^	0.016^*^
Digital maker education	0.039^**^	0.008	0.024^*^	0.027^**^
Community incentives (VMO)	0.223^***^	0.164^***^		0.117^***^
Community collaboration (VSY)	0.200^***^	0.145^***^		0.104^**^
Explanatory variables				
Virtual Community Job Embeddedness (VCJE)		0.304^***^	0.593^***^	
Community benefits (VEB)				0.122^***^
Job fit (VEF)				0.151^***^
Emotional links (VEL)				0.115^***^
R^2^	0.362	0.651	0.481	0.698
ΔR^2^		0.289^***^	0.119^***^	0.336^***^
F	143.233	336.101	194.769	364.601

*p < 0.1, **p < 0.05, **p < 0.01.

Model 4 replaces the overall construct of VCJE in Model 2 with its three specific dimensions: community benefits, job fit, and emotional links. The empirical results show that all three dimensions of VCJE have a significant positive impact on digital makers’ continuous participation intention (β₁ = 0.122, β₂ = 0.151, *β* = 0.115; all *p* < 0.01), thereby verifying Hypotheses H1, H2, and H3.

In addition, based on Models 1 and 3, a mean comparison test revealed that the impact of VCJE on continuous participation intention is significantly greater than the combined impact of community incentives and community collaboration (M = 0.170, t = 5.094). This demonstrates that the inclusion of VCJE provides strong predictive power for digital makers’ continuous participation intention.

Empirical analysis reveals that the job fits dimension exerts a significantly stronger effect on digital makers’ continuance participation intention than community benefits and emotional links. The core reason is that the essence of virtual digital maker communities is a work setting for value creation and capability monetization. Digital makers’ primary motivation for participating in such communities is to achieve precise alignment between their own interests, capabilities and platform tasks, thereby acquiring core resources for self-actualization and career development— a demand that takes precedence over derivative needs such as material gains and emotional bonds. Meanwhile, digital makers enjoy greater autonomy in behavioral choices in virtual settings. The experience of job fits characterized by temporal and spatial flexibility and capability compatibility directly reduces their participation costs and enhances their sense of gain from participation, thus driving continuance participation intention in a more direct and core manner.

## 6 Conclusions and discussion

This study draws the following key conclusions: ① VCJE in digital maker communities presents a three-dimensional structure comprising community benefits, emotional links and job fits, which constitutes a novel extension of job embeddedness in traditional work settings to the virtual community context. ② The measurement scale of VCJE consists of three factors with a total of 14 items, and data analysis demonstrates that this scale achieves an ideal level of reliability and validity. ③ VCJE, as a distinct construct independent of factors such as community incentives and community collaboration, exhibits strong explanatory power in predicting digital makers’ continuance participation intention in the community.

Job embeddedness in traditional organizations is rooted in physical, stable employment relationships and tangible work contexts, with its core lying in objective interest binding and physical network constraints. Its three dimensions—links, fits and sacrifice—all revolve around quantifiable material benefits, fixed relationships among organizational members and the adaptability to physical environments, emphasizing the objective constraints on employees’ turnover behavior (Mitchell et al., 2001). In contrast, the VCJE proposed in this study is grounded in the contractual cooperative relationships, borderless network characteristics and psychologically and emotionally driven interaction modes of virtual maker communities. Its essence is a collection of attractive forces for continuance participation formed between digital makers and virtual communities based on psychological identification, emotional links and value co-creation, with the core focusing on psychological resource appreciation and virtual network attachment.

The VCJE scale developed in this study represents an innovative reconstruction of traditional job embeddedness measurement tools, rather than a mere contextual adaptation. Its 14 items accurately capture the unique characteristics of virtual communities, and this scale can provide important support for future research on the impacts of non-work factors on digital makers’ behaviors in crowdsourcing communities, virtual communities and even digital communities in general. The forces emphasized by VCJE possess distinct virtual community attributes (Acar, 2019; Akhavan, 2021), which can offer valuable references and insights for research on digital makers’ behaviors such as continuance participation, knowledge sharing and value co-creation (Jabagi et al., 2019; Wong et al., 2021), as well as for studies on the organization, operation, governance and management of virtual communities (Vohra and Bhardwaj, 2019; Zhanbo et al., 2024).

The study finds that among the three dimensions of VCJE, job fit exerts a more prominent influence on digital makers’ continuance participation intention. This indicates that platforms should take the precise matching mechanism as the core of community operation: they need to leverage big data technology to build an intelligent matching system that connects digital makers’ capabilities and interests with platform projects, so as to achieve personalized and accurate matching between tasks and digital makers. Meanwhile, platforms should optimize project design and classification, take into account both the exertion of digital makers’ capabilities and their interest preferences, and create a flexible and adaptable work environment. By fundamentally enhancing digital makers’ participation experience, platforms can thereby strengthen their continuance participation intention.

### 6.1 Theoretical implications

The core theoretical contribution of this study lies in breaking the shackles of traditional job embeddedness theory, which has historically been confined to physical organizational contexts. In response to the unique attributes of virtual maker communities in the digital economy, this research reconstructed the conceptual connotation and dimensional system of job embeddedness. This study achieved an innovative extension of the theory across three dimensions—conceptual essence, theoretical extension, and predictive validity—rather than a mere contextual adaptation. Consequently, it provides a robust new theoretical framework and analytical perspective for organizational behavior research in digital and virtual job settings.

First, this study clarified the fundamental conceptual differences between VCJE and traditional organizational job embeddedness, which are reflected in three key areas: ① Shift in Linkages: The foundation of links has shifted from physical strong ties, such as human, financial, and material resources in traditional models, to virtual weak ties centered on psychology, emotion, and values (Hom et al., 2012; Acar, 2019). The objects of linkage have evolved from specific organizational members to the holistic field of mainstream communities. ② Evolution of Fit: The logic of fit has moved from long-term adaptation to physical environments and organizational values toward flexible matching of interests, capabilities, and spatio-temporal work arrangements (Xu et al., 2019; Acar, 2019; Akhavan, 2021). This shift weakens value-binding with task-releasing enterprises and strengthens psychological congruence between digital makers and their communities. ③ Transformation of Interest Orientation: The orientation has transformed from the perception of resource loss in the traditional “sacrifice” dimension to the perception of resource acquisition and appreciation in the “community benefits” dimension. By positioning positive gains—such as material rewards, psychological satisfaction, and capability enhancement—at the core of the construct, this model breaks away from the “resistance perspective” inherent in traditional frameworks (Kiazad et al., 2015). These fundamental differences render VCJE more compatible with digital virtual job settings, where formal employment relationships are absent and digital makers enjoy greater autonomy in behavioral choices (Markey and McIvor, 2018; González-Anta et al., 2021).

Second, this research reconstructed the analytical framework of job embeddedness, expanding its theoretical reach and applicable scenarios. Rather than simply applying traditional theory, this study reconstructed its framework based on the dual support of Conservation of Resources (COR) theory and network embeddedness theory (Kiazad et al., 2015). ① On one hand, it broke the fixed structure of “links, fit, and sacrifice” by reconstructing sacrifice into “community benefits,” thereby unifying all three dimensions under a positive perspective of “resource acquisition and appreciation”. This resolves the theoretical contradiction in traditional models where “sacrifice” relied on a resource loss perspective while other dimensions followed a gain logic (Coetzer et al., 2018; Ng and Feldman, 2013; Chen, 2022), thus improving the internal consistency of job embeddedness theory. ② On the other hand, it challenged the assumption of “objective factor dominance” by incorporating psychological, emotional, and virtual social resources into the core of the construct (González-Anta et al., 2021; Jabagi et al., 2019; Acar, 2019). By emphasizing these enduring subjective factors, the study addresses the theoretical deficiency regarding emotional factors in virtual contexts.

Third, this study verified the predictive validity of the new model for behavioral outcomes in virtual settings, extending the applicable boundaries of the traditional model. Traditional models primarily predict employee turnover in physical organizations based on objective constraints (Kiazad et al., 2020). However, in virtual maker communities, where the concept of “turnover” is blurred, the core behavioral outcome is continuance intention to participate (Ma et al., 2018; Wang et al., 2020). This study achieved two breakthroughs in predictive validity: ① Adaptation of Predictive Objects: The focus shifted from “turnover resistance” to “attractive forces for continuance participation”. The positive dimensions of community benefits, emotional links, and job fit align more closely with the participation motivations of digital makers—such as interest and resource acquisition (Wadhwani and Lubinski, 2025; Kherrazi, 2025)—rather than the logic of passive retention. ② Enhanced Explanatory Power: Empirical results demonstrated that after controlling for environmental variables (e.g., community incentives and collaboration) and individual characteristics (Sun et al., 2016), VCJE exerted a significant positive impact on continuance intention. Its explanatory power was found to be significantly higher than the combined effect of community incentives and collaboration.

### 6.2 Practical implications

First, the analysis of the connotations and characteristics of VCJE can be directly applied to the construction and management of virtual job environments. In the digital economy era, where “embeddedness is pivotal,” the “platform + community” operational model has become the mainstream paradigm for constructing virtual job settings. Platform developers and community managers should attach great importance to the construction and operation of the virtual social networks in which digital makers are embedded, thereby enhancing the networks’ enabling effects on makers’ proactive behaviors. In virtual communities such as crowdsourcing platforms, digital makers engage in project-based or gig work. While work-related factors still exert an influence on behaviors and performance, their impact is significantly weakened compared to traditional work settings, whereas the forces derived from non-work factors and social networks are substantially amplified. Therefore, platform enterprises should fully explore the potential of VCJE to drive digital maker engagement.

Second, the extension and innovation of the three dimensions of VCJE provide actionable guidance for the operation and management of crowdsourcing communities. To improve digital makers’ continuance intention to participate and further boost the vitality and innovation performance of the entire ecosystem, platform enterprises and community managers should enhance makers’ VCJE across the following three dimensions: ① Job Fit: Leverage big data analytics to accurately identify digital makers’ interests and skills, thereby achieving precise matching between maker demands and community services, incentive mechanisms, and project tasks. ② Emotional Links: Adopt diverse measures to foster a vibrant community atmosphere and strengthen makers’ socio-emotional sense of belonging. For instance, when the community scale is large, managers can segment the community based on maker characteristics to facilitate in-depth communication among members with shared interests and values. ③ Community Benefits: Enhance community stickiness by improving digital makers’ sense of gain and benefit, which effectively increases their switching costs for leaving the community. In practice, this requires providing systematic material and non-material support tailored to digital makers’ heterogeneous demand types and hierarchical needs.

Third, to enhance digital makers’ continuance intention to participate and their creativity in value co-creation, platform enterprises should increase investment in embeddedness mechanisms in addition to optimizing traditional incentive and collaboration mechanisms. ① From a Network Structure Perspective: Platform operators can use big data analytics to help digital makers embed themselves in optimal positions within the virtual network. This ensures a high degree of alignment in individual interests, competencies, and values with other network members, creating sufficient space for mutual learning, resource sharing, and value co-creation. ② From a Network Relationship Perspective: Platform enterprises can organize non-work social and cultural activities to promote mutual understanding and communication. These efforts strengthen the relational ties between digital makers and the community, thereby enhancing their psychological and emotional belonging. ③ From a Social Capital Perspective: Platform enterprises can offer activities such as experience sharing, digital maker training, and public welfare services. Such initiatives transform the community network into a vital channel for makers to acquire developmental and growth resources, further enhancing their overall sense of gain within the community.

### 6.3 Limitations and future research directions

While this study has achieved meaningful results in the conceptual development, scale construction, and empirical verification of VCJE, several limitations remain that leave room for further exploration. Cultural Specificity: First, all research samples were selected from domestic Chinese crowdsourcing and innovation platforms. Influenced by China’s collectivist culture, specific social attributes, and distinct platform operational models, the research conclusions may possess cultural biases. Consequently, they may not directly reflect the behavioral logic and embeddedness characteristics of digital makers within Western individualist cultures. Methodological constraints: Second, all data were collected via self-reported questionnaires, which are inherently susceptible to respondents’ subjective perceptions, social desirability bias, and recall bias. Such biases may lead to an overestimation of the relationships between variables and might fail to fully and objectively capture the actual state of digital makers’ VCJE and their behavioral decision-making mechanisms. Generalizability of scenarios: Third, the generalizability of the findings is limited by the study’s focus on crowdsourcing and enterprise-led virtual innovation communities, which feature distinct project orientations and interest-based connections. When extending these conclusions to other digital communities, such as knowledge-sharing or interest-exchange platforms, researchers must consider adaptability issues arising from differences in community attributes. Lack of cross-platform comparison: Fourth, this study did not conduct a comparative analysis with mainstream Western gig economy platforms. Given the significant differences between Chinese and Western platforms regarding operational models, group composition, institutional environments, and cultural backgrounds, empirical cross-cultural and cross-platform tests are still lacking to verify the adaptability of these conclusions to Western gig economy scenarios.

Future research can be advanced across four key areas: Cross-Cultural Comparison: Conduct comparative studies by selecting samples from representative Chinese and Western gig economy platforms to explore the moderating effects of cultural contexts on the dimensional structure and mechanisms of VCJE. Methodological triangulation: Adopt mixed-method research designs that combine objective behavioral data with subjective questionnaire data. This approach will mitigate the limitations of self-reported data and enhance the credibility of the research conclusions. Cross-scenario validation: Expand research samples to diverse types of digital communities to test the cross-scenario adaptability of the VCJE construct and its measurement scale. Universal theoretical modeling: Construct a cross-platform theoretical model of VCJE. This should clarify the construct’s applicable boundaries and adjustment paths under various institutional and cultural backgrounds, providing a universal theoretical reference for the operation of global digital platforms.

## Data Availability

The original contributions presented in the study are included in the article/supplementary material, further inquiries can be directed to the corresponding author.
